# Nursing in Egypt: exploring the link between work centrality and work-related well-being: a cross-sectional study

**DOI:** 10.1186/s12912-026-04613-z

**Published:** 2026-04-17

**Authors:** Safaa Hassan Zaki Abbas, Ashraf A. Noah, Iman A. F. Aboelsaad

**Affiliations:** Alexandria Clinical Research Administration. Alexandria Directorate of Health Affairs, Ministry of Health and Population of Egypt, Alexandria, Egypt

**Keywords:** Work centrality, Nurses, Work-related well-being, Pleasure in work, Health, Relationships with colleagues, Egypt

## Abstract

**Background:**

Work centrality is defined as the importance individuals attribute to their work role. Its relationship with different dimensions of work-related well-being among nurses remains underexplored, particularly in the Egyptian healthcare context. This study aimed to examine the association between work centrality and work-related well-being among Egyptian nurses.

**Methods:**

A cross-sectional survey was conducted among nurses in healthcare units affiliated with the Ministry of Health and Population, Alexandria, Egypt. Validated questionnaires were translated into Arabic and distributed online to measure Work centrality and the three dimensions of work-related well-being: pleasure in work, health, and relationships. Factors associated with work centrality were examined using univariate linear regression models. Multiple linear regression assessed the associations between work centrality and well-being dimensions while adjusting for age, sex, marital status, education, work setting, and department. Potential effect modifications by sex, work setting, education level, and shift type were also explored.

**Results:**

A total of 342 nurses responded to the questionnaire with a response rate of 29.7%. Among the 337 respondents with complete data, 91% were females, and 91% were hospital-based nurses. Lower work centrality scores were more common among younger, married nurses, and those with graduate education, with a median score [Min, Max] of 21.0 [11.0, 31.0]. In univariate models, lower pleasure in work, poorer recovery, and weaker relationships with colleagues were significantly associated with lower work centrality (*p* < 0.001). In the adjusted models, work centrality remained positively associated with pleasure in work [B = 0.31, 95% CI 0.21–0.41, *p* < 0.001], need for recovery (better health/lower fatigue) [B = 0.20, 95% CI 0.10–0.31, *p* < 0.001], and relationships with colleagues [B = 0.22, 95% CI 0.11–0.34, *p* < 0.001]. Working shifts modified the associations between work centrality and both pleasure and health, but not relationships with colleagues.

**Conclusion:**

Higher work centrality was positively associated with greater workplace pleasure, better health (lower fatigue), and stronger collegial relationships among Egyptian nurses. These findings suggest that perceiving work as meaningful and central to professional identity may contribute to multiple dimensions of nurses’ well-being. Longitudinal studies are needed to confirm these associations.

**Trial registration:**

Clinical trial number: Not applicable.

**Supplementary Information:**

The online version contains supplementary material available at 10.1186/s12912-026-04613-z.

## Background

Employee well-being has hit crisis levels worldwide, with recent data showing unprecedented burnout rates, diminished job satisfaction, and increasing mental distress rooted in their professional environment [1]. Work-related well-being refers to the total quality of an employee’s experiences and performance at work [2, 3]. The three main components of well-being include psychological (happiness), social (relationships), and physical (health) well-being [3]. Work-related well-being is influenced by factors such as professional growth, work‒life balance, stress management, job satisfaction, and positive relationships with colleagues [4]. While high well-being fosters a sense of empowerment and appreciation, its absence triggers a cascade of negative organizational outcomes, most notably in the nursing profession [5, 6]. 

In Egypt, the nursing workforce faces a profound occupational health crisis characterized by chronic shortages, excessive workloads, and low job satisfaction, mirroring the global trends but intensified by local system challenges [7–9]. Recent studies document a high nurse-to-patient ratio (1:10 in public hospitals), inadequate staffing, extended shifts, and disproportionately low financial compensation, driving burnout, fatigue, and intention to migrate abroad among graduates [10, 11]. The World Health Organization’s State of the World’s Nursing 2025 report underscores Egypt’s position in the Eastern Mediterranean Region with a nurse density of just 15.5 per 10,000 population, compounded by health strains such as severe fatigue in 66–77% of cases and a poor work environment overlooking nurses’ well-being [12].

The clinical and organizational significance of nurse well-being cannot be overstated. From a clinical perspective, work-related well-being of nursing staff is directly correlated with patient safety, medication errors, and overall quality of care [13–15]. A study conducted in Tabriz, Iran, highlights that higher nurse happiness levels significantly reduce burnout, leading to a measurable decrease in nursing errors and improved patient safety [16]. Furthermore, a better collegial nurse-physician relationship and a work environment supporting nurse’s well-being is associated with lower medication errors and healthcare-associated infections [14, 17]. Additionally, broader investigations into nurses’ health indicate that nurses with poor mental or physical health are significantly more likely to commit clinical errors than their healthier colleagues [14, 18]. 

From an organizational standpoint, poor nurse well-being causes significant financial and operational challenges. Psychological distress poses a greater threat to productivity through absenteeism, presenteeism, and increased turnover [4]. A recent study involving 400 Egyptian nurses highlights this crisis, where 66.7% reported poor psychological well-being, which was significantly correlated to absenteeism, presenteeism, impaired daily activities, and massive productivity losses [4]. In 2023, a study in Saudi Arabia estimated that annual absenteeism-induced costs were $4 million, with an average monthly loss of 0.62 workdays per nurse primarily due to various psychosocial pressures [19]. 

Enhancing staff well-being is a central focus within hospital strategic planning, in the face of rising patient demand and insufficient staffing levels [20]. The World Health Organization has highlighted the need for multi-level strategies to promote healthcare workers’ well-being [21]. Various interventions have been implemented internationally, ranging from individual-level resilience training and mindfulness-based stress reduction to organizational changes like flexible scheduling, improved nurse-to-patient ratio, leadership training, and organizational support programs [22, 23]. However, the individual’s work-related values and psychological orientations may play a critical role in shaping how nurses perceive stressors and respond to supportive interventions [24]. Hence, many of these strategies fail to account for the internal value systems, such as work centrality.

**Fig. 1:**
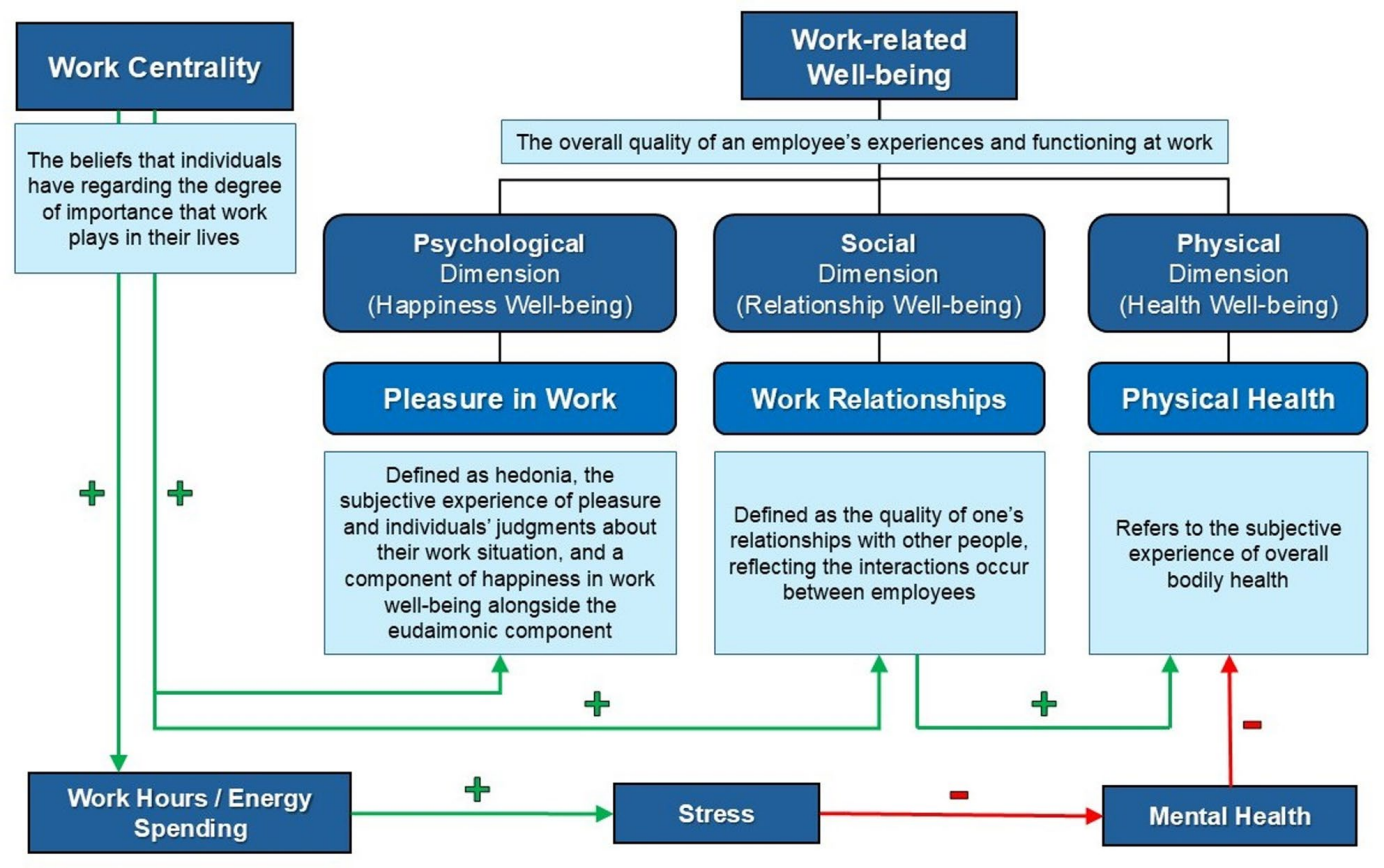
Theoretical framework for work centrality and work-related well-being dimensions

Work centrality is a cognitive construct referring to the degree of importance that work has in the life of an individual at any given point in time [25, 26]. In the nursing context, work centrality captures the relative importance of the nursing role relative to other life roles, such as family, leisure, and religion [27]. Contemporary healthcare organizations increasingly favor “ideal workers” who exhibit high work centrality, prioritizing professional obligations over personal life [28]. While high work centrality is often positively correlated with organizational commitment and job satisfaction [29], its relationship with work-related well-being is non-linear and complex, as illustrated in the theoretical framework adopted from Wang [30]. (Fig. 1).

For some, a high degree of work identification provides a sense of purpose that buffers against burnout; for others, it leads to work-life interferences and physical exhaustion [31]. The relationship between work centrality and work-related well-being in nursing is an underexplored area. While external factors like salary, workload, and social support are indeed vital to well-being [6, 32] the internal prioritization of work (Centrality) may determine whether a nurse views a challenging shift as a rewarding professional duty or an intrusive life stressor. Understanding this relationship is particularly urgent in Egyptian healthcare contexts. Egyptian nurses operate under unique, compounding pressures, creating a high-stress environment where the nurses struggle to separate their professional duties from personal well-being [33, 34]. Limited research focused on well-being and work centrality, and pertains to Western cohorts, which may not translate to the cultural and economic realities of the Middle East [30, 35]. 

### Theoretical framework

#### Core constructs

We adopted the theoretical framework previously described by Wang et al., (Fig. 1) [30]. Work centrality, defined as the perceived importance of work as a life domain, acts as a primary predictor, where high centrality amplifies positive well-being outcomes (e.g., pleasure derived from meaningful tasks) but heightens vulnerability to fatigue when resources like supportive relationships are depleted [25, 36]. Warr’s model posits well-being as multidimensional: pleasure in work aligns with affective components (job satisfaction), health/fatigue with anxiety-contentment and depression-enthusiasm continua, and colleague relationships as environmental vitamins (e.g., skill use, social contact) that prevent resource loss [37]. 

#### Pathways and moderation

Conservation of Resources (COR) [38], theory explains the linkage: high work centrality motivates resource investment (e.g., time/energy), fostering pleasure and relationships when gains occur, but triggering burnout/fatigue amid losses like overload, common in nursing contexts. Empirical paths include direct effects (centrality leads to pleasure via identity fulfillment) and indirect ones (centrality leads to fatigue mediated by relational quality) [39, 40]. 

#### Nursing application

In Egyptian nursing, this framework hypothesizes that low centrality (due to poor conditions) erodes well-being. By exploring these dynamics, this study aims to examine the association between work centrality and specific dimensions of well-being (happiness, relationships, and health) and provide a nuanced understanding of how Egyptian nurses balance their professional identity with their personal health and social stability. Such insights are essential for developing targeted, culturally sensitive organizational interventions that go beyond wellness programs in the Egyptian nursing workforce.

## Materials and methods

### Study objectives

To examine the association between work centrality and well-being among Egyptian nurses and explore how work centrality is related to the three dimensions of work-related well-being.

### Study setting and population

This study was conducted in hospitals and family medicine units (FMUs) affiliated with the Alexandria Directorate of Health Affairs under the Ministry of Health and Population (MOHP) of Egypt. These settings provide a wide range of low-cost services to the residents of Alexandria and the neighboring governorates. The setting encompasses 15 affiliated hospitals (providing secondary and tertiary care) and eight health districts comprising over one hundred primary care units.

The participants pool consisted of professional nurses representing two distinct clinical environments: (1) primary care nurses, based in FMUs and focusing on community health, preventive services, and outpatient management, (2) hospital-based nurses, employed in general and specialized hospitals, and managing high acuity cases, emergency trauma, and acute clinical interventions.

### Study design

An anonymous online cross-sectional survey was conducted via a structured questionnaire via a social media platform (WhatsApp). The link to the questionnaire was circulated among nurses who were registered at the MOHP databases of Alexandria Directorate of Health Affairs, during the data collection period from October 1st, 2023, to December 30th, 2023. All study procedures adhered to the ethical principles of the Declaration of Helsinki and were reported in accordance with the STROBE guidelines for cross-sectional studies. Nurses who agreed to participate in the survey consented online before completing the survey form.

A minimum sample size of 280 participants was required to power the multiple linear regression models to detect an adjusted R-square of 5% at a significance level of 5% and a power of 80% while controlling for all 7 covariates.

### Data collection tools

The survey included the following sections:

In Section 1, we collected participant demographics, including sex and age (stratified into distinct categories: < 30 years, 30–39 years, 40–49 years, and ≥ 50 years). Marital status was defined as single, divorced, married, or widowed. Additionally, participants’ work settings were defined as either primary care or hospital settings, with their respective working departments categorized as medical or administrative. Educational attainment was elucidated, distinguishing between graduate degrees (Bachelor’s, Master’s, and PhD) and undergraduate degrees obtained from nursing high schools, with durations of 3 and 5 years. The participants’ professional experience was categorized into discrete intervals: less than 5 years, 5–10 years, 11–20 years, and more than 20 years. Furthermore, working shifts were classified as either rotating shifts or fixed morning shifts. 

Section 2 assessed our exposure of interest: *Work centrality* measured by a 7-item questionnaire (Appendix 1) [41, 42]. This modified version, which was validated by Wang et al. [30]., is a shorter version of the original 12-item scale developed by Paullay et al., in 1994 [43]. Construct and discriminant validity as well as reliability of the original scale, were demonstrated in several studies [30]. 

Although Wang et al., used Principal Component Analysis and initially revealed a two-factor structure (explaining 45.92% and 16.85% of the variance), likely a method effect caused by positive and negative item wording, the combined 7-item scale demonstrated strong overall internal consistency (alpha = 0.797). Consequently, the instrument was treated as a single, unidimensional measure, with all items aggregated to yield a comprehensive work centrality score.

The responses were collected on a 5-point Likert scale ranging from 1 (strongly disagree) to 5 (strongly agree), where higher scores indicate a stronger belief in the centrality of work in one’s life. Three items were reverse-scored (“Work should only be a small part of one’s life”, “I have other activities more important than my work”, and “To me, my work is only a small part of who I am”). A total score, ranging from 7 to 35, was computed by summing responses, with higher scores indicating a stronger belief that work is central to the participant’s life.

*Work-related well-being* was assessed using selected subscales of the “Questionnaire Experience and Assessment of Work” questionnaire, abbreviated from Dutch as VBBA 2.0 [30, 44]. The original instrument (VBBA) was developed and validated by Van Veldhoven and Meijman (1994) [44]. VBBA is a well-established instrument widely used in occupational health research to measure psychosocial work pressure and employee well-being [30]. The reduced VBBA 2.0 versions showed better quality [30]. We adopted the scales of VBBA 2.0 used by Wang et al., measuring work-related well-being [30]. The selected VBBA 2.0 scales (Appendix 2): “pleasure in work”, “need for recovery”, and “relationship with colleagues” subscales assessed 3 distinct dimensions: happiness well-being, health well-being, and relationship well-being, respectively. The instrument demonstrated satisfactory psychometric properties, including clear factor structures and acceptable internal consistency coefficients across subscales (generally α > 0.70) [30]. The pleasure in work subscale has demonstrated good internal consistency (Cronbach’s α = 0.825), the need for recovery and the relationships with colleagues subscales showed acceptable reliability, with Cronbach’s alpha of 0.761 and α = 0.699, respectively [30]. 

*Happiness well-being* was measured by the “pleasure in work” scale, which consists of four items. Responses were given on a 5-point Likert scale ranging from 1 (strongly disagree) to 5 (strongly agree). (Appendix 2). The item “Today, I did my work because I had to, and that says it all” was reverse-scored [30]. The total score ranges from 4 to 16, with higher scores indicating greater happiness at work.

*Health well-being* was measured by the “Need for Recovery” (NFR) scale in VBBA 2.0 to test the stress level and physical fatigue after work. On the NFR scale, three items were answered via a 5-point Likert scale. All Items were reversed. Thus, higher total scores, which range from 3 to 15, indicate higher levels of health and lower fatigue [30]. 

*Relationship well-being* was measured by the “relationships with colleagues” scale of the VBBA 2.0. The participants responded to the 4-item scale on a 5-point Likert scale, with higher total scores, ranging from 4 to 16, indicating better relationships with colleagues. The item “Did you have conflicts with your colleagues today?” was reverse-scored. The numerical responses of all the questions were summed for each scale and subscale to generate continuous scores [30]. 

### Translation and adaptation

All tools were translated into the official Arabic language by two professional translators. A panel of five academic experts subsequently evaluated the content validity and linguistic accuracy of the translated tools. Each expert independently assessed the instruments on the basis of item relevance, comprehensiveness, and clarity. To increase accuracy and mitigate potential threats to the study’s validity, some items were refined for clarity before being back-translated into English by linguists. The final translated version showed good internal consistency, where Cronbach’s alpha coefficients for the need for recovery, relationship with colleagues, and pleasure in work subscales were 0.85, 0.77, and 0.71. respectively. The overall well-being modified VBBN 2.0 scale yielded a reliability of 0.59, while the work centrality scale was 0.70.

### Data collection

The online survey prompt included the study’s aim and a link to the survey, along with instructions to guide participants in completing it. Frequent reminders were sent to encourage nurses to complete the survey. The form received responses for three months. To prevent duplicate submissions, participants could complete the survey only once. The optimal time to complete the questionnaire was 10–15 min.

### Statistical analysis

Participant characteristics were summarized via descriptive statistics for the whole sample and across work centrality score tertiles. Differences in participants’ characteristics across work centrality tertiles were examined via the chi-square test or Fisher’s exact test for categorical variables and the Kruskal‒Wallis test for continuous data. Univariate associations between participant characteristics and well-being outcomes (pleasure in work, need for recovery, and relationships with colleagues) were assessed via linear regression models after pertinent assumptions were tested. The work centrality score and the three well-being subscores were standardized so that the effect estimate (standardized beta) represents the change in standard deviation (SD) of well-being subscores for each one-SD increase in work centrality.

Multiple linear regression models were constructed to adjust for potential confounding variables, including age, sex, marital status, education level, work setting, and department. Experience levels were removed from the models because of high multicollinearity with age (VIF = 24.0). Effect modifications between work centrality and well-being subscores by sex, work setting, education level and working shifts were explored. All the statistical analyses were performed via R version 4.3.1 (R Foundation for Statistical Computing, Vienna, Austria), with the statistical significance level set at *p* < 0.05.

## Results

### Characteristics of the study population

Among the 1150 nurses working in selected settings, 342 participants completed the questionnaire with a 29.7% response rate. Of the 342 participants, 337 provided complete data and were included in the analysis. The sample was predominantly female (91%), with nearly half under 30 years old (45%) and married (55%) (Table 1). Most worked in hospital settings (91.1%) and medical departments (97%) rather than administrative jobs, and approximately two-thirds lacked an academic degree. Compared to females, male participants were younger and less experienced (Supplemental Table 1). All males were hospital-based and more likely to hold academic degrees and less likely to work fixed morning shifts. The population characteristics across work centrality tertiles are described in (Supplemental Table 2).

**Table 1:** Overall study population characteristics and work centrality tertiles

	Overall (*N* = 337) 21.0 [11.0, 31.0]	Work Centrality			
		1st tertile (*N* = 113) 18.0 [11.0, 20.0]	2nd tertile (*N* = 112) 21.0 [20.0, 23.0]	3rd tertile (*N* = 112) 25.0 [23.0, 31.0]	*P* value*
**Male sex** (n, %)	32 (9.5%)	5 (4.4%)	16 (14.3%)	11 (9.8%)	0.032
**Age** (n, %)					
< 30 years	152 (45.1%)	67 (59.3%)	51 (45.5%)	34 (30.4%)	0.002
30–39 years	71 (21.1%)	23 (20.4%)	20 (17.9%)	28 (25.0%)	
40–49 years	69 (20.5%)	16 (14.2%)	28 (25.0%)	25 (22.3%)	
≥ 50 years	45 (13.4%)	7 (6.2%)	13 (11.6%)	25 (22.3%)	
**Marital status** (n, %)					
Single	131 (38.9%)	57 (50.4%)	39 (34.8%)	35 (31.3%)	0.022
Divorced	11 (3.3%)	6 (5.3%)	3 (2.7%)	2 (1.8%)	
Married	186 (55.2%)	48 (42.5%)	67 (59.8%)	71 (63.4%)	
Widowed	9 (2.7%)	2 (1.8%)	3 (2.7%)	4 (3.6%)	
**Primary care setting** (n, %)	30 (8.9%)	7 (6.2%)	10 (8.9%)	13 (11.6%)	0.566
**Medical Department** (n, %)	327 (97.0%)	110 (97.3%)	107 (95.5%)	110 (98.2%)	0.531
**Graduate education** (n, %)	110 (32.6%)	53 (46.9%)	31 (27.7%)	26 (23.2%)	0.001
**Experience** (n, %)					
< 5 years	89 (26.4%)	42 (37.2%)	25 (22.3%)	22 (19.6%)	< 0.001
5–10 years	89 (26.4%)	36 (31.9%)	34 (30.4%)	19 (17.0%)	
11–20 years	49 (14.5%)	15 (13.3%)	14 (12.5%)	20 (17.9%)	
> 20 years	110 (32.6%)	20 (17.7%)	39 (34.8%)	51 (45.5%)	
**Rotating Shifts**	288 (85.5%)	101 (89.4%)	96 (85.7%)	91 (81.3%)	0.391

* p values were generated via the chi-square test, Fisher’s exact test or the Kruskal‒Wallis test

### Associations between work centrality and well-being dimensions

**Fig. 2:**
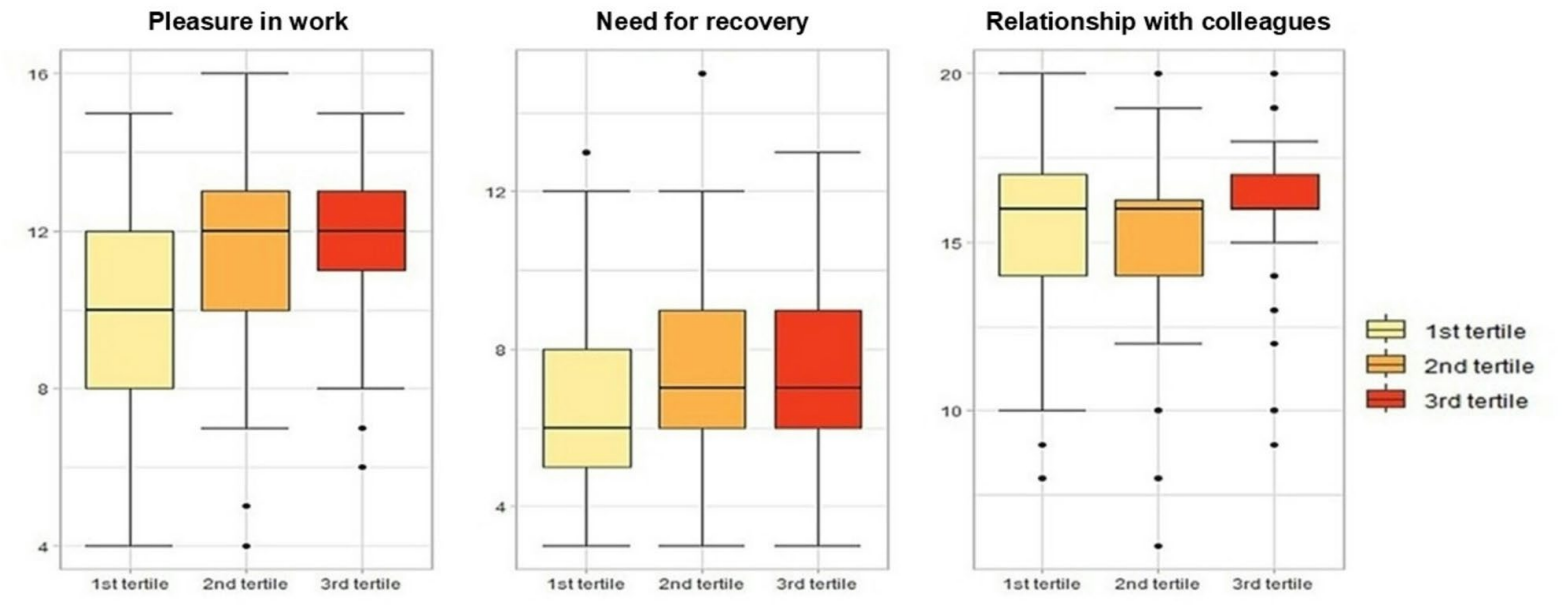
Distribution of well-being subscores across tertiles of work centrality

Overall, the median work centrality score [Min, Max] was 21.0 [11.0, 31.0] (Table 2). Lower scores of pleasure in work and need for recovery (indicating worse health/ higher fatigue) were significantly associated with lower work centrality scores (*p* < 0.001). While the median score relationship with colleagues remained consistent across different work centrality tertiles, lower levels of work centrality were associated with a wider range of scores (Fig. 2). Similar patterns were observed in female participants, whereas no significant associations were found in males (Supplemental Table 2).

**Table 2:** Overall well-being subscores by work centrality tertiles

	Work Centrality*				*P* value†
	Overall (*N* = 337) 21.0 [11.0, 31.0]	Tertile1 (*N* = 113) 18.0 [11.0, 20.0]	Tertile 2 (*N* = 112) 21.0 [20.0, 23.0]	Tertile 3 (*N* = 112) 25.0 [23.0, 31.0]	
**Happiness in work** Median [Min, Max]	12.0 [4.00, 16.0]	10.0 [4.00, 15.0]	12.0 [4.00, 16.0]	12.0 [6.00, 15.0]	< 0.001
**Need for recovery** Median [Min, Max]	7.00 [3.00, 15.0]	6.00 [3.00, 13.0]	7.00 [3.00, 15.0]	7.00 [3.00, 13.0]	< 0.001
**Relationship with colleagues** Median [Min, Max]	16.0 [6.00, 20.0]	16.0 [8.00, 20.0]	16.0 [6.00, 20.0]	16.0 [9.00, 20.0]	0.004

Expressed as the median [Min, Max], **†***p* values are estimated from the Kruskal‒Wallis test

In univariate associations with linear regression models, the three well-being subscores were significantly associated with work centrality (Table 3, Supplemental Table 3). Work centrality contributed the most to the variability of pleasure in work *(R*^*2*^ = 13.2%), followed by need for recovery *(R*^*2*^ = 6.7%) and relationships with colleagues (*R*^*2*^ =3.6%) (Table 3). Pleasure in work scores were significantly higher among middle-aged nurses (30–39 years) [standardized B 0.46 (95% CI, 0.23 to 0.64); *p* < 0.001]. Married and widowed participants presented higher scores of pleasure in work than did the singles. Nurses working in primary care reported greater pleasure in work scores than those in hospital settings did. Those with higher levels of education and working rotating shifts reported lower levels of pleasure in work scores [standardized B -0.34, 95% CI -0.57 to -0.12; *p* = 0.003] and [standardized B -0.35 (95% CI, -0.64 to -0.04); *p* = 0.025], respectively. Similarly, the need for recovery scores showed similar associations except with working rotation shifts (Supplemental Table 3). The relationship with colleagues’ scores was not associated with any of the covariates (Supplemental Table 3).

**Table 3:** Association of well-being subscores with work centrality in univariate and adjusted models

	Unadjusted Standardized Beta (95%CI)	*P* value	*R* ^2^	Adjusted Standardized Beta (95%CI)*	*P* value	Adjusted *R*^2^
Happiness in work	0.37 (0.27–0.47)	< 0.001	0.132	0.31(0.21–0.41)	< 0.001	0.210
Need for recovery	0.26 (0.16–0.37)	< 0.001	0.067	0.20 (-0.10-0.31)	< 0.001	0.182
Relationship with colleagues	0.20 (0.09–0.30)	< 0.001	0.036	0.22 (0.11–0.34)	< 0.001	0.060

*Adjusted Beta from fully adjusted models, controlling for age, sex, marital status, work setting, department, education and working shifts

**Fig. 3:**
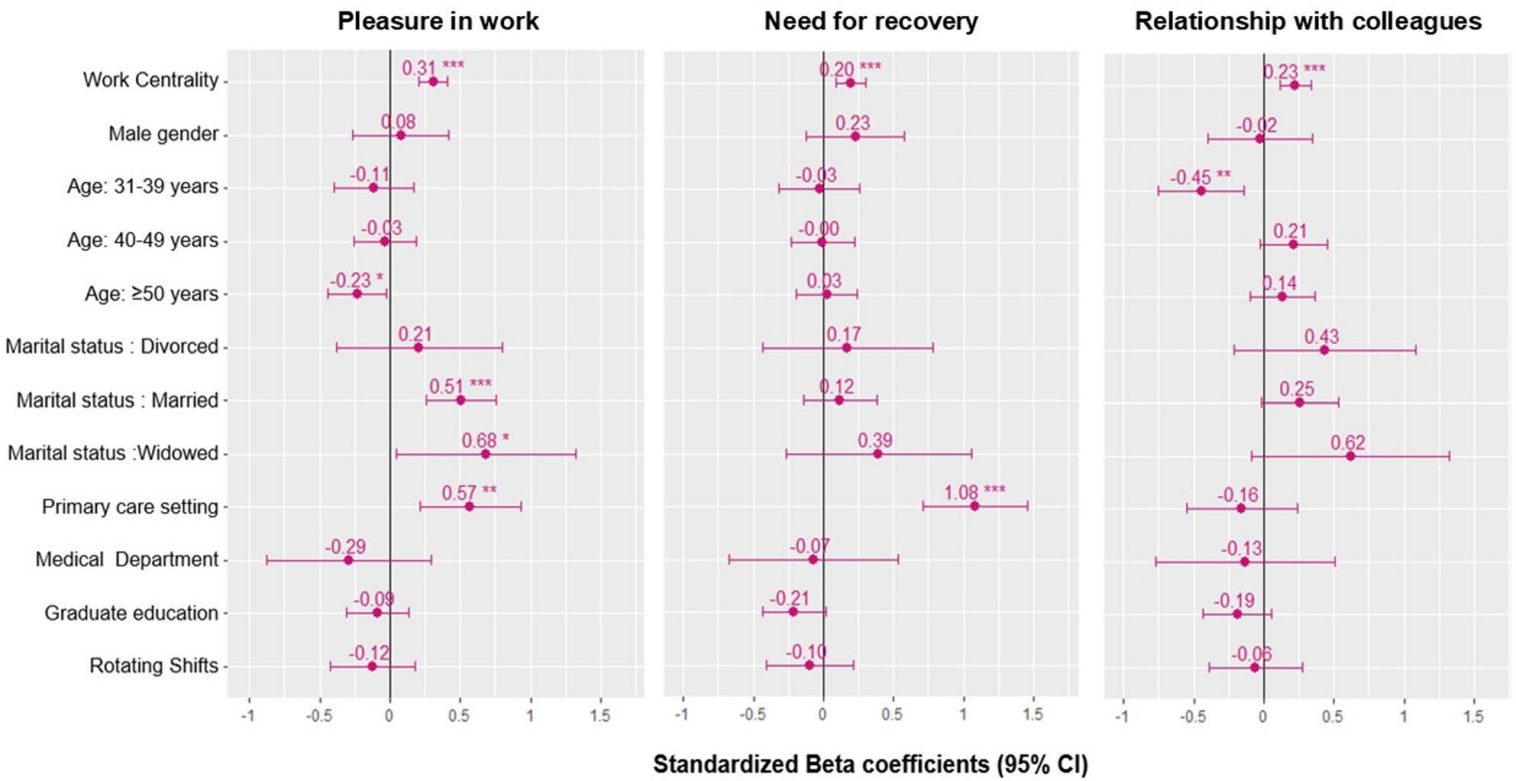
Adjusted associations of well-being subscores with work centrality

In the adjusted models, the three well-being scores remained positively associated with work centrality (Fig. 3; Table 3). For each 1-SD increase in the work centrality score, the standardized pleasure in work, need for recovery, and relationship with colleagues scores were significantly higher by 0.31 SD (95% CI, 0.21 to 0.41; *p* < 0.001), 0.20 SD (95% CI, 0.10 to 0.31; *p* < 0.001), and 0.22 SD (95% CI, 0.11 to 0.34; *p* < 0.001), respectively.

The associations with most factors remained significant except for education level, and working shifts were no longer significant in multivariable models (Supplemental Table 4). Married and widowed status relative to single status similarly remained significantly associated with pleasure in work only [standardized B 0.51 (95% CI, 0.25 to 0.76), *p* < 0.001] and [B 0.68 (95% CI, 0.03 to 1.33), *p* = 0.039], respectively. The primary care setting was significantly associated with a higher need for recovery score (better health/lower fatigue) [standardized B 1.17 (95% CI, 0.79 to 1.55); *P* < 0.001], whereas all other associations were weakened. The 30–39 years age group was significantly associated with a lower score for relationships with colleagues [standardized beta − 0.45 (95% CI, -0.76 to -0.13), *p* = 0.005] (Supplemental Table 4).

### Effect modification of the associations between work centrality and well-being dimensions by sex, work setting, education level, and shift type

A statistically significant effect modification by working shifts was observed in the association between pleasure in work and work centrality (*p* = 0.018) (Supplemental Table 5). The association was significant only for nurses working rotating shifts [standardized B 0.35 (95% CI, 0.24 to 0.46)] (Supplemental Table 5). Similarly, a significant effect modification by shift category was detected in the association between need for recovery and work centrality (*p* = 0.002). Among those on rotating shifts, the need for recovery score is positively associated with work centrality [Standardized B 0.27 (95% CI, 0.16 to 0.38)]. In contrast, among those on morning shifts, higher work centrality is associated with lower levels of need for recovery scores (worse health/ higher fatigue) [standardized B -0.17 (95% CI, 0.47 to 0.13)] (Supplemental Table 5). No effect modification by education level was detected. A significant interaction was seen with the work setting in the association between work centrality and relationships with colleagues (*p* = 0.002). The association was stronger in primary care settings [standardized B 0.84 (95% CI, 0.29 to 1.38)] than in hospital settings [standardized B 0.18 (95% CI, 0.07 to 0.30)] (Supplemental Table 5).

## Discussion

**Fig. 4:**
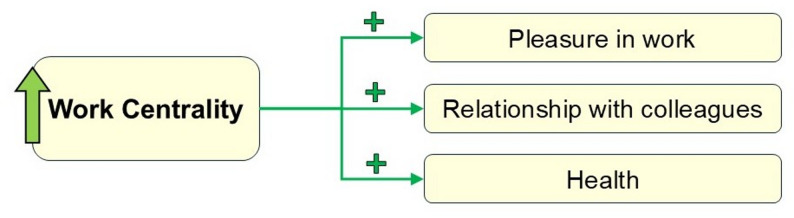
Work centrality associations with pleasure at work, health, and relationship with colleagues

Given that nurses constitute the largest group of healthcare professionals and are essential to quality care, patient safety, and resource management, understanding how work centrality impacts their well-being is crucial [45, 46]. In our study of 337 nurses affiliated with the Ministry of Health and Population in Egypt, lower work centrality was more prevalent among younger and married nurses, as well as those holding graduate degrees. Overall, higher work centrality scores were positively associated with greater workplace pleasure, better health and lower need for recovery, and stronger relationships with colleagues (Fig. 4), with more pronounced associations among nurses working rotating shifts. These findings align with foundational occupational psychology theories, notably Warr’s Vitamin model and Paullay et al., work centrality framework, and prior research identifying work centrality as an important correlate of nurses’ pleasure at the workplace, health, and collegial relationship well-being [36, 37, 43]. 

Health well-being and pleasure at work are closely interconnected. Excessive workload and stress can reduce the level of pleasure experienced in the workplace, while a lack of hedonia may also negatively affect an individual’s physical health status [47]. In the present study, higher work centrality was associated with better health well-being and greater pleasure at work. This finding contrasts with previous research suggesting that higher work centrality may be linked to greater fatigue [25] and increased stress, which in turn reduces workplace pleasure [45]. This discrepancy may be explained by the possibility that strong work centrality functions as a motivational resource by fulfilling intrinsic needs like meaning and competence, Thus, individual’s invest more energy in valued tasks, encouraging professional achievement and helping to counterbalance the negative psychological effects of stress and overexertion [35, 43]. Accordingly, healthcare institutions should foster organizational climates that strengthen professional identity, recognition, and engagement, enabling work centrality to serve as a protective factor rather than a source of strain.

Furthermore, previous research has indicated that individuals with high work centrality may prioritize work-related interactions for networking-building and support seeking, potentially at the expense of other personal relationships [35]. Congruently, our findings revealed a positive association between work centrality and relationships with colleagues, which was more pronounced in primary care settings. One possible explanation is that primary care units typically operate with smaller and more stable teams, fostering repeated interactions, interpersonal familiarity, and the development of trust among staff [48]. Such conditions may strengthen collegial relationships, particularly among individuals who place greater importance on their work and are therefore more engaged in collaborative team dynamics [49, 50]. 

Organizing working shifts has a considerable influence on nurses’ work-related well-being [51]. In our study, the positive association between work centrality and both workplace pleasure and health well-being was stronger among nurses working rotating shifts compared to those on fixed morning schedules. Rotating shifts are frequently associated with sleep disruption, irregular routines, and increased physical and emotional strain [52]. Under these demanding conditions, nurses who perceive their work as central and meaningful may cope more effectively with schedule-related stress [53]. In this context, work centrality may function as a buffering factor, reducing the burden of rotating shift demands and helping sustain motivation, emotional stability, and recovery. This buffering mechanism may explain the more pronounced associations observed among nurses working rotating shifts. Yet, given that literature examining the relationship between shift patterns and well-being presents mixed and context-sensitive results, further research is warranted to confirm or refute the modifying effect of rotating shifts on the association between work centrality and both workplace pleasure and health well-being [54]. 

The aforementioned findings necessitated a comprehensive exploration of the traits that might affect the concept of work centrality itself. We observed that lower work centrality was more prominent among younger and married nurses. This observation is consistent with previous studies indicating that younger nurses (Millennials and Generation Z) prioritize work-life balance and demonstrate professional orientations that differ from those of older cohorts [55]. While they seek professional fulfillment and career achievement, they tend to adopt effective time-management strategies to structure work demands in ways that prevent professional responsibilities from overwhelming their personal lives. Consequently, their professional orientation tends to integrate career aspirations with other important life domains, including family commitments, personal well-being, and social engagement, rather than emphasizing a predominantly work-centered identity [55]. In parallel, married nurses may experience additional familial responsibilities and emotional demands that redistribute attention across life domains, potentially reducing the centrality of work within their overall identity [36, 56]. Hence, further research is recommended to explore the unique personal traits of younger generations to establish supportive work environments that enhance their productivity.

Surprisingly, graduate education was associated with lower work centrality. This finding may reflect a broader professional identity among highly educated nurses. Nurses who pursue advanced degrees often engage in academic, administrative, research, or leadership roles in addition to clinical practice. As a result, direct bedside care may represent only one component of their overall professional identity. Therefore, lower work centrality in this subgroup may not indicate reduced commitment, but rather a redistribution of professional focus across multiple roles and career pathways [57]. These findings suggest that healthcare institutions should recognize and support the multifaceted professional roles of highly educated nurses by providing structured career pathways that integrate clinical, academic, and leadership responsibilities.

Finally, the current study underscores the importance of work centrality as a factor associated with nurses’ well-being, including higher workplace pleasure, better health (lower fatigue), and relationship well-being. Future longitudinal research is recommended to further investigate these associations to enhance strategies for supporting nurses’ well-being in diverse healthcare settings.

### Strengths and limitations

The current study has several notable strengths, as it addresses an important issue in healthcare by exploring the impact of work centrality on nurses’ well-being, using well-established scales for measuring key variables. Additionally, the inclusion of diverse factors and the application of robust statistical methods enhanced the validity and reliability of the study.

However, several limitations should be acknowledged. The cross-sectional design hinders causal inference, and we cannot exclude the possibility of reverse causation. Recruitment via WhatsApp and snowball online distribution may have introduced selection bias. The use of self-reported measures raises the possibility of misclassification bias. Finally, we excluded professional experience from the adjusted regression models due to severe multicollinearity with age. Hence, we could not assess the associations of work centrality with years of experience, which reflect accumulated clinical competence and professional maturity that are conceptually distinct from biological age. Our findings only apply to nurses in a similar governmental context.

## Conclusion

The current study demonstrates that work centrality is positively associated with psychological well-being, health well-being, and improved collegial relationships among Egyptian nurses. The associations appear stronger within rotating shift contexts and primary care settings, suggesting that perceiving work as meaningful and central to one’s professional identity may function as a contextual resilience factor.

Our findings highlight the importance of fostering meaningful professional engagement while ensuring supportive working conditions. Interventions aimed at strengthening professional identity, recognition, and participatory work environments may contribute to improved nurse well-being. However, longitudinal and multi-center studies are needed to clarify causal pathways and determine how work centrality interacts with career development, shift structures, and evolving professional roles.

## Supplementary Information

Below is the link to the electronic supplementary material.

Supplementary Material 1

## Data Availability

The datasets generated and/or analysed during the current study are not publicly available due to restrictions imposed by the Egyptian Ministry of Health and Population but are available from the corresponding author on reasonable request.

## Abbreviations

MOHP: Ministry of Health and Population of Egypt
FMUs: Family medicine units
CI: confidence interval
NFR: Need for Recovery
SD: Standard deviation
