# Supplementary material for: Nursing in Egypt: exploring the link between work centrality and work-related well-being: a cross-sectional study
Source: BMC Nurs. 2026 Apr 17;25:377. doi: 10.1186/s12912-026-04613-z (PMC13094161; doi:10.1186/s12912-026-04613-z)
Supplement: Supplementary file 1 — Supplementary Material 1 [file 12912_2026_4613_MOESM1_ESM.docx]

***Supplementary Material***

**Nursing in Egypt: Exploring the Link between Work Centrality and Work-Related Well-Being: A Cross-sectional Study**

**Appendix 1. Work Centrality Scale**

**Appendix 2. The selected scales of the VBBA 2.0 questionnaire measuring work-related well-being**

**Supplemental Table 1. Participants’ characteristics by gender**

**Supplemental Table 2. Association of wellbeing scores with work centrality tertiles by gender category**

**Supplemental Table 3. univariate associations of wellbeing subscores with work centrality and other covariates**

**Supplemental Table 4. Adjusted Associations of wellbeing scores with work centrality and other covariates**

**Supplemental table 5. Effect modification of the wellbeing subscores association with work centrality by gender, work setting, education level and working shifts.**

***Appendix 1***

**Work Centrality Scale**

| **Strongly disagree** | **Disagree** | **Neutral** | **Agree** | **Strongly agree** | **Work Centrality Scale** |
| --- | --- | --- | --- | --- | --- |
|  |  |  |  |  | 1- Work should only be a small part of one’s life |
|  |  |  |  |  | 2- In my view, an individual’s personal life goals should be work oriented. |
|  |  |  |  |  | 3-The major satisfaction in my life comes from my work |
|  |  |  |  |  | 4-The most important things that happen to me involve my work. |
|  |  |  |  |  | 5-I have other activities more important than my work. |
|  |  |  |  |  | 6-Work should be considered central to life. |

**Appendix 2.**

**The selected scales of the VBBA 2.0 questionnaire measuring work-related well-being**

| **Strongly disagree** | **Disagree** | **Neutral** | **Agree** | **Strongly agree** | **Work-related well-being** |
| --- | --- | --- | --- | --- | --- |
|  |  |  |  |  | ***Pleasure in your work***  1-Today, I found my work stimulating |
|  |  |  |  |  | 2. Today, I did my work because I had to, and that says it all. |
|  |  |  |  |  | 3. Today, I enjoyed my work. |
|  |  |  |  |  | 4. Today, I continually had to overcome my resistance in order to do my work |
|  |  |  |  |  | ***Need for recovery***  1-Today, I find it difficult to relax |
|  |  |  |  |  | 2. I felt worn out. |
|  |  |  |  |  | 3. I felt rather exhausted because of my job. |
|  |  |  |  |  | ***Relationships with colleagues***  1-Today, could you, if necessary, ask your colleagues for help? |
|  |  |  |  |  | 2. Today, did you get on well with your colleagues? |
|  |  |  |  |  | 3. Did you have conflicts with your colleagues today? |
|  |  |  |  |  | 4. Was there a good atmosphere between you and your colleagues today? |

**Supplemental Table 1: Participants’ characteristics by gender**

|  | Female  (N=303) | Male  (N=32) | Overall  (N=335) |
| --- | --- | --- | --- |
|  |  |  |  |
| Age |  |  |  |
| Less than 30 | 133 (43.9%) | 19 (59.4%) | 152 (45.4%) |
| 31-39 years | 61 (20.1%) | 10 (31.3%) | 71 (21.2%) |
| 40-49 years | 64 (21.1%) | 3 (9.4%) | 67 (20.0%) |
| Older than 50 years | 45 (14.9%) | 0 (0%) | 45 (13.4%) |
| Marital Status |  |  |  |
| Bachelor | 117 (38.6%) | 14 (43.8%) | 131 (39.1%) |
| Divorced | 11 (3.6%) | 0 (0%) | 11 (3.3%) |
| Married | 166 (54.8%) | 18 (56.3%) | 184 (54.9%) |
| Widowed | 9 (3.0%) | 0 (0%) | 9 (2.7%) |
| Hospital Setting | 275 (90.8%) | 32 (100%) | 307 (91.6%) |
| Medical Department | 296 (97.7%) | 30 (93.8%) | 326 (97.3%) |
| Graduate education | 90 (29.7%) | 20 (62.5%) | 110 (32.8%) |
| Experience |  |  |  |
| Less than 5 years | 79 (26.1%) | 10 (31.3%) | 89 (26.6%) |
| 5-10 years | 74 (24.4%) | 15 (46.9%) | 89 (26.6%) |
| 11-20 years | 45 (14.9%) | 4 (12.5%) | 49 (14.6%) |
| More than 20 years | 105 (34.7%) | 3 (9.4%) | 108 (32.2%) |
| Rotating shifts | 258 (85.1%) | 30 (93.8%) | 288 (86.0%) |

**Supplemental Table 2: Association of wellbeing scores with work centrality tertiles by gender category**

|  |  | Work centrality | | |  |
| --- | --- | --- | --- | --- | --- |
| Male |  |  |  |  |  |
|  | **Overall**  (N=32)  22.0 [16.0, 30.0] | **1st tertile**  (N=5)  18.0 [16.0, 19.0] | **2nd tertile**  (N=16)  21.0 [21.0, 23.0] | **3rd tertile**  (N=11)  24.0 [23.0, 30.0] | ***P*-value*** |
| Happiness in work, *Median [Min, Max]* | 12.0 [4.00, 15.0] | 10.0 [8.00, 14.0] | 12.0 [4.00, 15.0] | 11.0 [7.00, 14.0] | 0.581 |
| Need for recovery, *Median [Min, Max]* | 7.00 [3.00, 12.0] | 6.00 [4.00, 10.0] | 8.00 [3.00, 12.0] | 7.00 [5.00, 10.0] | 0.573 |
| Relationship with colleagues, *Median [Min, Max]* | 16.0 [10.0, 20.0] | 16.0 [13.0, 19.0] | 15.5 [10.0, 18.0] | 16.0 [13.0, 20.0] | 0.602 |
| Female |  |  |  |  |  |
| Work centrality, *Median [Min, Max]* | **Overall**  (N=305)  21.0 [11.0, 31.0] | **1st tertile**  (N=108)  18.0 [11.0, 20.0] | **2nd tertile**  (N=96)  21.0 [20.0, 23.0] | **3rd tertile**  (N=101)  25.0 [23.0, 31.0] | ***P*-value** |
| Happiness in work, *Median [Min, Max]* | 12.0 [4.00, 16.0] | 10.0 [4.00, 15.0] | 12.0 [5.00, 16.0] | 12.0 [6.00, 15.0] | <0.001 |
| Need for recovery, *Median [Min, Max]* | 7.00 [3.00, 15.0] | 6.00 [3.00, 13.0] | 7.00 [3.00, 15.0] | 7.00 [3.00, 13.0] | <0.001 |
| Relationship with colleagues, *Median [Min, Max]* | 16.0 [6.00, 20.0] | 16.0 [8.00, 20.0] | 16.0 [6.00, 20.0] | 16.0 [9.00, 20.0] | 0.005 |

*P values were generated from Kruskal Wallis tests

**Supplemental Table 3: univariate associations of wellbeing subscores with work centrality and other covariates**

|  | **Work Happiness** | | **Need for recovery** | | **Relationship with colleagues** | |
| --- | --- | --- | --- | --- | --- | --- |
|  | **Standardized Beta (95%CI)** | **P value** | **Standardized Beta (95%CI)** | **P value** | **Standardized Beta (95%CI)** | **P value** |
| **Work centrality** | 0.37 (0.26, 0.46) | <0.001 | 0.26 (0.16, 0.36) | <0.001 | 0.20 (0.10, 0.31) | <0.001 |
| **Male gender** | 0.03 (-0.34, 0.39) | 0.87 | 0.07 (-0.29, 0.44) | 0.689 | 0.06 (-0.30, 0.43) | 0.722 |
| **Age category** |  |  |  |  |  |  |
| 30-39 years | 0.46 (0.23, 0.69) | <0.001 | -0.37 (0.14, 0.61) | 0.001 | -0.13 (-0.37, -0.10) | 0.265 |
| 40-49 years | -0.12 (-0.35, 0.11) | 0.297 | -0.03 (-0.26, 0.20) | 0.786 | 0.17 (-0.07, 0.40) | 0.157 |
| < 50 years | -0.15(-0.38, 0.07) | 0.117 | 0.07 (-0.15, 0.30) | 0.532 | 0.14 (-0.09, 0.38) | 0.223 |
| **Marital status** |  |  |  |  |  |  |
| Divorced | 0.26 (-0.32, 0.84) | 0.376 | 0.36 (-0.24, 0.97) | 0.238 | -0.01 (-0.64, 0.60) | 0.955 |
| Married | 0.64 (0.43, 0.86) | <0.001 | 0.35 (0.12, 0.56) | 0.002 | 0.02 (-0.20, 0.25) | 0.809 |
| Widowed | 1.00 (0.36, 1.65) | 0.002 | 0.87 (0.20, 1.53) | 0.011 | 0.30 (-0.37, 0.99) | 0.371 |
| **Setting primary care** | 0.83 (0.47, 1.20) | <0.001 | 1.27 (0.92, 1.62) | 0.034 | -0.12 (-0.50, 0.25) | 0.519 |
| **Medical Department** | -0.30 (-0.93, 0.33) | 0.335 | -0.12 (-0.75, 0.51) | 0.704 | -0.02 (-0.65, 0.61) | 0.952 |
| **Graduate Education** | -0.34 (-0.57, -0.12) | 0.003 | -0.42 ( -0.65,  -0.02) | <0.001 | -0.22 (-0.45, 0.01) | 0.057 |
| **Experience** |  |  |  |  |  |  |
| 5-10 years | 0.46 (0.26, 0.65) | <0.001 | 0.33 (0.17, 0.54) | <0.001 | -0.12 (-0.32, 0.08) | 0.233 |
| 11-20 years | 0.06 (-0.16, 0.28) | 0.592 | 0.03 (-0.18, 0.25) | 0.758 | -0.00 (-0.23, 0.22) | 0.985 |
| > 20 years | -0.16 (-0.39, 0.08) | 0.191 | -0.15 (-0.39, 0.08) | 0.218 | -0.14 (-0.39, 0.10) | 0.243 |
| **Rotating shifts** | -0.35 (-0.64, -0.04) | 0.025 | -0.29 (-0.59, 0.01) | 0.06 | 0.07 (-0.24, 0.37) | 0.666 |

**Supplemental Table 4: Adjusted Associations of wellbeing scores with work centrality and other covariates**

|  | **Pleasure in work** | | **Need for recovery** | | **Relationship with colleagues** | |
| --- | --- | --- | --- | --- | --- | --- |
|  | **Standardized Beta (95%CI)** | **P value** | **Standardized Beta (95%CI)** | **P value** | **Standardized Beta (95%CI)** | **P value** |
| **Work centrality** | 0.31 (0.21, 0.41) | <0.001 | 0.20 (0.1, 0.31) | <0.001 | 0.22 (0.11, 0.34) | <0.001 |
| **Male gender** | 0.07 (-0.27, 0.42) | 0.668 | 0.22 (-0.12, 0.57) | 0.207 | -0.02 (-0.40, 0.35) | 0.898 |
| **Age category** |  |  |  |  |  |  |
| 30-39 | -0.12 (-0.41, 0.16) | 0.402 | -0.06 (-0.35, 0.23) | 0.684 | -0.45 (-0.76,  -0.13) | 0.005 |
| 40-49 | -0.04 (-0.27, 0.18) | 0.724 | -0.03 (-0.26, 0.20) | 0.777 | 0.21 (-0.03, 0.46) | 0.088 |
| < 50 | -0.24 (-0.46,  -0.03) | 0.028 | -0.01 (-0.23, 0.21) | 0.948 | 0.14 (-0.09, 0.37) | 0.242 |
| **Marital status** |  |  |  |  |  |  |
| Divorced | 0.20 (-0.39, 0.79) | 0.507 | 0.15 (-0.46, 0.75) | 0.629 | 0.43 (-0.21, 1.08) | 0.188 |
| Married | 0.51 (0.25, 0.76) | <0.001 | 0.12 (-0.14, 0.38) | 0.358 | 0.25 (-0.02, 0.53) | 0.071 |
| Widowed | 0.68 (0.03, 1.33) | 0.039 | 0.36 (-0.30, 1.02) | 0.286 | 0.63 (-0.08, 1.33) | 0.081 |
| **Primary care Setting** | 0.59 (0.21, 0.96) | 0.002 | 1.17 (0.79, 1.55) | <0.001 | -0.17 (-0.58, 0.24) | 0.415 |
| **Medical Department** | -0.34 (-0.96, 0.28) | 0.285 | -0.14 (-0.77, 0.50) | 0.671 | -0.18 (-0.86, 0.50) | 0.606 |
| **Graduate Education** | -0.09 (-0.31, 0.14) | 0.445 | -0.21 ( -0.43, 0.02) | 0.072 | -0.19 (-0.43, 0.05) | 0.124 |
| **Rotating shift** | -0.013 (-0.44, 0.18) | 0.401 | -0.15 (-0.47, 0.16) | 0.345 | -0.05 (-0.39, 0.29) | 0.785 |

***Need for recovery was reversed scored (higher socre indicate better health)**

**Supplemental table 5: Effect modification of the wellbeing subscores association with work centrality by gender, work setting, education level and working shifts.**

|  | **Gender** | | | **Work setting** | | | **Education** | | | **Shifts** | | |
| --- | --- | --- | --- | --- | --- | --- | --- | --- | --- | --- | --- | --- |
|  | **Male** | **Female** | **P-int** | **Hospital** | **Primary care** | **P-int** | **Graduate** | **Undergrad** |  | **Rotating** | **Morning** |  |
| **Pleasure in work** | 0.59  (0.11, 1.07) | 0.28  (0.17, 0.39) | 0.218 | 0.33  (0.22, 0.43) | 0.32  (-0.01, 0.65) | 0.337 | 0.31  (0.08, 0.54) | 0.28  (0.16, 0.39) | 0.381 | 0.35  (0.24, 0.46) | 0.01  (-0.26, 0.27) | 0.018 |
| **Need for recovery** | 0.47  (0.09, 0.84) | 0.16  (0.04, 0.28) | 0.495 | 0.12  (0.11, 0.33) | 0.40  (-0.10, 0.91) | 0.864 | 0.24  (0.05, 0.43) | 0.14  (0.00, 0.28) | 0.252 | 0.27  (0.16, 0.38) | -0.17  (-0.47, 0.13) | 0.002 |
| **Relationship with colleagues** | 0.32  (-0.10, 0.75) | 0.21  (0.09, 0.33) | 0.506 | 0.18  (0.07, 0.30) | 0.84  (0.29, 1.38) | 0.002 | 0.35  (0.01, 0.45) | 0.23  (0.09, 0.36) | 0.747 | 0.21  (0.08, 0.33) | 0.19  (-0.17,0.54) | 0.930 |
